# Then there were seven**:** a commentary on creating a public involvement strategy group for a policy research unit in behavioural science

**DOI:** 10.1186/s40900-023-00413-w

**Published:** 2023-02-04

**Authors:** Dave Green, Val Bryant, Stuart Edwards, Caroline Kemp, Maisie McKenzie, Sudhir Shah, Irene Soulsby

**Affiliations:** Policy Research Unit Behavioural Science, Population Health Sciences Institute, Newcastle University, Baddiley-Clark Building, Newcastle upon Tyne, NE2 4AX England, UK

**Keywords:** Public involvement, Behavioural Science, Health and social care, Policy research

## Abstract

The National Institute for Health and Care Research (NIHR) Policy Research Unit in Behavioural Science (PRU-BS) was funded to inform government on the application of behavioural science in health and social care policy. What makes this unit different to other topic specific ones, was the wide range of its brief. Because of this, the PPI group would need to include a wide range of experience and expertise and be prepared to learn. We were a different type of public group for a different type of task. This paper deals with how we approached this. In this paper we outline how the PPI plan in the funding proposal for the PRU-BS was adapted to real world challenges. We describe the stages in the formation of the PPI Strategy Group and how a virtual platform was created to ensure good communication. We discuss our pragmatic approach of developing Terms of Reference and a PPI strategy document. Given the restrictions imposed by the Covid-19 pandemic we explain how we tackled PPI SG member induction sessions, meetings and training sessions. To illustrate how the group operates we provide an example of our involvement in a PRU-BS project. Central to our paper is the lessons we learned. We hope the challenges we met in forming the unique PPI SG, how these were overcome, and our recommendations will help others faced with a similar task.

## Plain English summary

The Policy Research Unit in Behavioural Science (PRU-BS) was formed in early 2019, funded by the National Institute for Health and Care Research (NIHR). The aim of the unit is to advise the government on the use of behavioural science (the study of human behaviour) to inform health and social care policy. From the outset the aim was to embed PPI in all aspects of the unit’s work from the governance and direction of the unit to the individual research projects it conducts. As behavioural science cuts across all aspects of health, recruiting members of the public to work within the PRU-BS required careful thought. In this paper we describe the processes of recruiting to our PPI Strategy Group, the induction and training, and the ways in which we worked to develop the group and become embedded within the unit. Lastly, we present several recommendations based on our experiences of forming the PPI Strategy Group. Although this is aimed primarily at those contemplating setting up a group whose remit extends beyond a single research project, we hope this will be a useful resource for the public, researchers and others working in PPI.

## Background

**Fig. 1:**
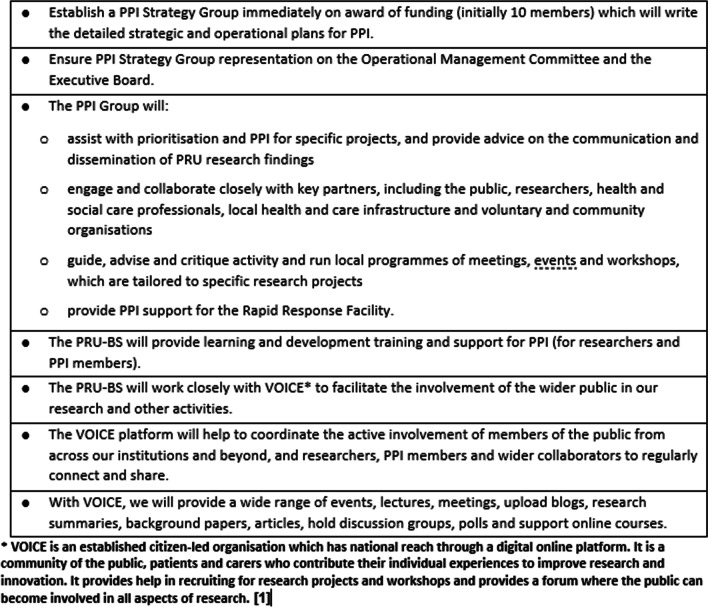
Proposed plan for PPI from the application for PRU funding

Behavioural science covers a range of disciplines and in the context of public health, influences almost everything, bringing science to bear on all aspects of human behaviour. In a research unit with a behavioural science focus how do you ensure meaningful PPI and where do you begin? This was the challenge faced by the Policy Research Unit in Behavioural Science (PRU-BS), a collaboration between Newcastle University, the University of Warwick, University College London and the London School of Hygiene and Tropical Medicine, funded in December 2018 by the National Institute for Health and Care Research (NIHR). The first author (DG), who was known to the lead applicant through his involvement in NIHR funding panels, was involved in the development of the application for the unit’s funding (Fig. 1). The application proposed that PPI, along with equality and diversity, be cross-cutting themes and embedded in all aspects of the PRU from its governance to individual project design and delivery, to dissemination and impact.

The PRU-BS was established in early 2019, its aim to inform public policy on health, health systems and preventing ill health using behavioural science evidence, theory and methods to support decision making [2]. For such a unique unit we would require a unique PPI team prepared to take a much more comprehensive view of health and social care, moving out of their comfort zones. This would demand that public members were open minded, flexible and prepared to engage with a wide range of health and social care challenges. At the beginning this was a daunting prospect. We needed to recruit members of the public who not only had the varied life and PPIE experiences, a professional attitude and the ability to fully participate in formal unit meetings, but could also provide a public perspective on proposed projects [3]. The behavioural science focus and the wide remit of the unit also demanded that public members were prepared to actively seek out new experiences and acquire new skills. This meant not only a willingness to undergo training but also a commitment to be proactive in disseminating the work of the group.

Much of the published literature relates to the experiences of researchers and public in forming PPI groups for a single project [4–12]. There is little regarding PPI groups whose role is to input into a number of projects over a long period, rapid response to research ideas and oversight of the management at a strategic level. Two studies were identified [13, 14] though both were topic specific, and the breadth of the research was not as extensive as in our own unit. We could not identify any published literature regarding PPI in behavioural science.

This paper describes in chronological order our experience of implementing the proposed PPI plan in a newly established policy research unit, in particular the objective of creating a PPI strategy group (PPI SG), and the lessons learned which we believe will be of benefit to others in establishing a PPI group whose remit is broader than the involvement in a single project. It highlights the tensions between the understandable optimism of a funding application and what is possible in practice. In the following sections we first outline the behind-the-scenes activities and resources required to implement the PPI plan. Next, we describe the function of the PPI SG within the unit. The final section provides some reflections on the process and what we learned.

## Steps to ensure public representation in the unit

From the very beginning PPI was considered essential in all aspects of the unit’s work and conduct. Although this is a given, we needed to make it a reality. The funding application stated that the creation of a PPI group would be an early priority though only one PPI member (DG) had been recruited as the PPI lead. It was evident that without the support of an administrative team, to facilitate planning and the budget, little could be done to form a PPI group. Also, the unit did not reach its full complement of researchers until the summer of 2019. A public group must have something to do, a long hiatus between selection and engagement with the work of the unit could impact negatively on motivation and morale. It was necessary to keep the momentum going.

Things began to progress when a member of the PRU-BS research team with experience of working with PPI groups took on the role of PPI liaison/mentor to support, advise and provide updates on the progress of establishing the unit. In addition, once there was support with administration and finance from a new unit manager, the PPI lead, mentor and the PRU-BS director could now discuss ideas for the PPI element and draw up plans for implementation.

It was decided to proceed slowly with the plan to create a PPI SG but recruit a core group to enable initial planning. We needed to recruit the right people with the right skills and attitudes who would appreciate the sensitivity of work with government bodies. This was achieved initially by a process called ‘shoulder tapping’ where someone with a known background and track record in PPI is approached to join a group. This ensures that recruits are of known quality and reliability. The downside of course is that recruitment is restricted to people known to us and limits opening out to a more diverse population. In the autumn of 2019, a co-lead (IS)- who was known to DG—was appointed following an informal interview with the PPI lead, PPI mentor, unit manager and the director. Their role was to support the current PPI lead (DG) and ensure coverage of the PRU-BS meetings. This was followed closely by informal interviews with, and recruitment of, two further members of the public (VB and CK) with PPI experience who were known to members of the PRU-BS. These four members formed the core PRU-BS PPI SG. There was an opportunity for the group to get to know each other, and PRU-BS researchers, at the official PRU-BS launch in January 2020.

## How we share and communicate

It was evident that if the PPI SG was to communicate effectively, then a robust mechanism to facilitate interaction and exchange was needed. The use of a virtual platform was included in the initial funding proposal. Negotiations began early with VOICE (based at UK's National Innovation Centre for Ageing at Newcastle University), to create a virtual platform. This platform would enable PPI SG members to communicate with each other and the unit researchers. It was considered an evolving innovation which would adapt to the needs of its users. There were some challenges in developing a platform which satisfied both PRU-BS and the VOICE team. Technical and communication issues took time to resolve, and we were grateful for the input of the PRU-BS unit manager and administrator who liaised with the VOICE team. So far, the platform comprises a page containing the project plain English summaries, a general discussion group where members can ask or pose questions and an archive and library of articles, open access publications and training materials. Access to parts of the platform are restricted to PRU-BS researchers and the PPI SG. We expect it will evolve to meet the needs of users, and eventually will give the public access to the proposals and published policy briefs with a chance to comment.

## Producing terms of reference and the PPI Strategy document

Once we had the initial PPI SG in place, we needed to understand its’ purpose, activities and progression. As indicated in the PRU funding applied our first task was to draw up Terms of Reference and a Strategy Document to guide the group. Rather than trying to invent the wheel we based our Terms of Reference [15] on an example used by the NIHR. Similarly, we obtained the NIHR School for Public Health Research’s PPI Strategy which served the purpose of providing an initial framework and template for our own document. We amended this in line with the requirements of the PRU-BS. Our PPI strategy document [16] was developed by DG and IS with the guidance of the PPI mentor. The document was circulated to other PRUs who suggested some minor changes. The PPI Strategy objectives draw heavily on the UK standards for public involvement and engagement in research [17]. Each objective has action plans, milestones, outputs and timelines. We expect the strategy document to evolve in response to the wider needs of the PPI SG and the unit.

## Expanding and ensuring diversity of the group

Because of the specific remit of the unit, we had to make sure we had the right people in our PPI SG. Members required not only a wide range of experiences but also a willingness to learn and be adaptable and most importantly be able to respond quickly to requests for input when required. It was decided at a very early stage that the group should be as diverse as possible in respect of experience, skills, ethnicity, gender and age. Our early members were all based in the Northeast of England (one of the disadvantages of ‘shoulder tapping’) and we wished to include people from other regions. Now that the PRU-BS administrative structure was in place the real job of expanding the PPI SG, and ensuring diversity, could begin. VOICE, which offers opportunities for involvement in research to a national audience, was used to advertise for new PPI SG members. Advertising for the PPI SG through VOICE provided a large pool of potential candidates but it was not possible to target members with specific demographic characteristics. This was addressed through the shortlisting process. Nineteen candidates eventually applied and four were shortlisted and interviewed using Zoom in mid-August 2020. Those who were not successful joined a PRU-BS PPI sub-group through the Voice platform and agreed to be involved when the need arose.

**Fig. 2:**
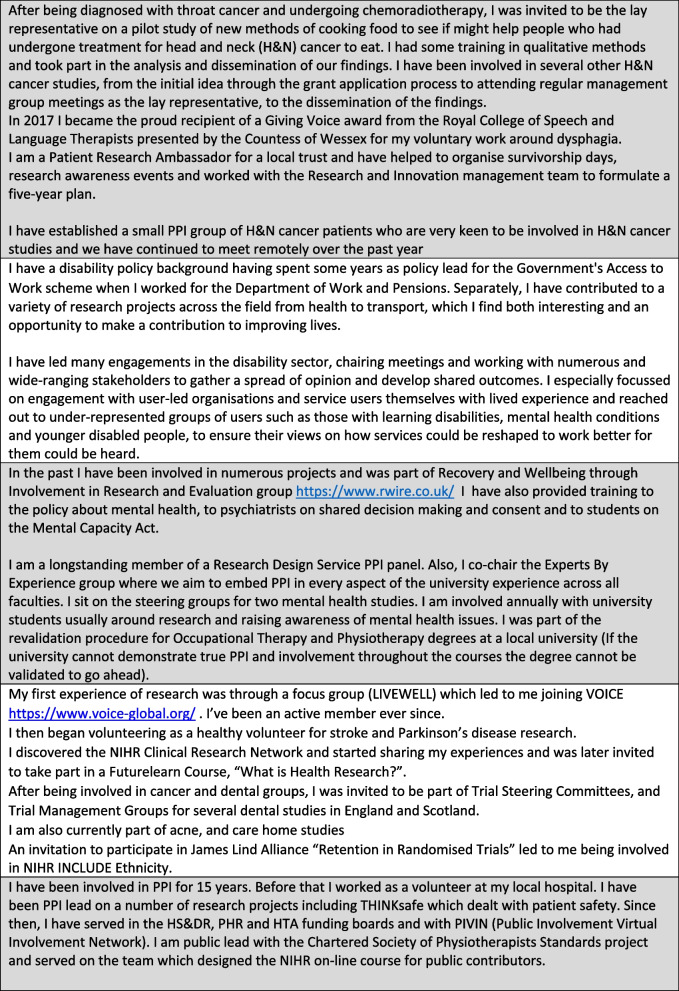

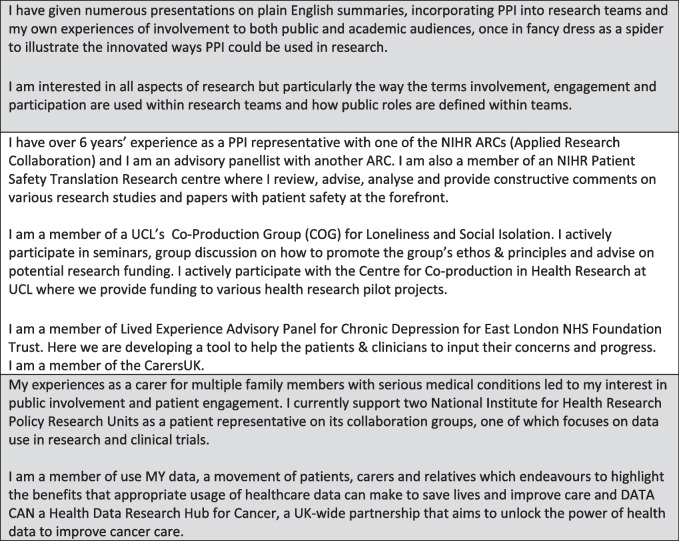
PPI biographies for the current PPI Strategy Group members

The interview panel consisted of the PPI co-leads (IS and DG), PPI mentor and the unit manager. Of the four candidates interviewed three proceeded to join the group. Whilst the initial plan was to recruit ten members it was felt that the PPI SG now included seven members with a diverse ethnic and geographical spread, younger age and range of skills and experience. There were no initial or subsequent tensions between diversity and usefulness, all SG members were selected first on the skills and experience based criteria outlined earlier. Figure 2 includes short biographies of all PPI SG members.

## Inducting group members into the unit

We were aware that the PPI SG faced a substantial learning curve, in the understanding of behavioural science and its implications for health care and while the group had been selected for their experience and flexibility there was a lot to learn. We dealt with this partly through ongoing training, but it was essential that we had a shared understanding of the remit of the unit and the group’s role. The approach to this task is summed up by a group member.

"I wondered if I should be in the PRU for mental health as this is where my interest lies, but then I realised that while my knowledge of behavioural science was limited, I later came to understood that behavioural science is the most important influencer of all and if you have that framework, you can apply it to benefit every situation."

It was key that SG members learned about the role of behavioural science in health policy. We planned induction sessions for our complete PPI SG. These would normally take place over one day and be conducted face to face. However, we needed to face the realities of Covid-19 and two shorter (two hours) induction sessions were held via Zoom over two days in the last two weeks of September 2020. These sessions were an important introduction to the work of the unit. The PRU-BS director gave a presentation about the unit and how behavioural science was an integral part of the research. Research leads gave an overview of their projects and took questions afterwards. The PPI lead also gave an insight into PPI and how this would work within the unit. It is absolutely crucial that group members get on with each other. This cohesiveness and ability to work together effectively is the difference between success and failure. There was time at the end to chat more generally and it was obvious that the right choice of members had been made as the group got on well together. This socialising is important not only for the cohesiveness of the group but provides the opportunity for members to share their experiences, skills, and knowledge.

## How we work

### Meetings and training

The onset of the COVID-19 pandemic presented challenges for PPI SG. Issues included replacing face to face meetings and subsequent training with a virtual platform (Zoom). However, the use of Zoom did enable more frequent direct communication with researchers about their projects. One difficulty was the lack of devices (laptops/tablets) among members which was resolved by the provision of new ones. We were fortunate that the PPI for the PRU-BS was well funded and acknowledge that funding is essential to ensure good quality PPI. Adequate funding was an area the director considered of prime importance to the effectiveness of the unit. All members of the PPI SG are reimbursed for any time they spend on unit activities at INVOLVE/NIHR rates. We also offer to pay for attendance at conferences related to, and also outside of, the unit remit to enable the personal development of members.

All PPI SG members meet monthly at a day and time that is convenient to them. We decided to restrict the meetings to one hour and keep them as informal as possible. The frequency of the meetings has been reviewed and members preferred to continue to meet monthly. There is an informal agenda (with items collated beforehand) and it is also a forum to discuss and identify training needs, for researchers to present or seek input into projects and, if time permits, to run short training sessions.

The PRU-BS hold monthly Management Meetings for all unit staff which the PPI SG co-leads attend. PPI is a standing agenda item, and the public lead submits a PPI update beforehand for circulation to all attendees. The PPI co-leads provide a verbal update, take questions and contribute to discussions regarding other agenda items. One PPI co-lead attends the bi-annual Scientific Advisory Board -a panel of independent professionals and a public member who offer advice and guidance to the PRU—again providing a written update beforehand and verbally in the meeting and takes questions from the board. To ensure the rest of the PPI SG are kept up to date the PPI co-leads provide feedback on both these meetings at the PPI SG meeting.

**Fig. 3:**
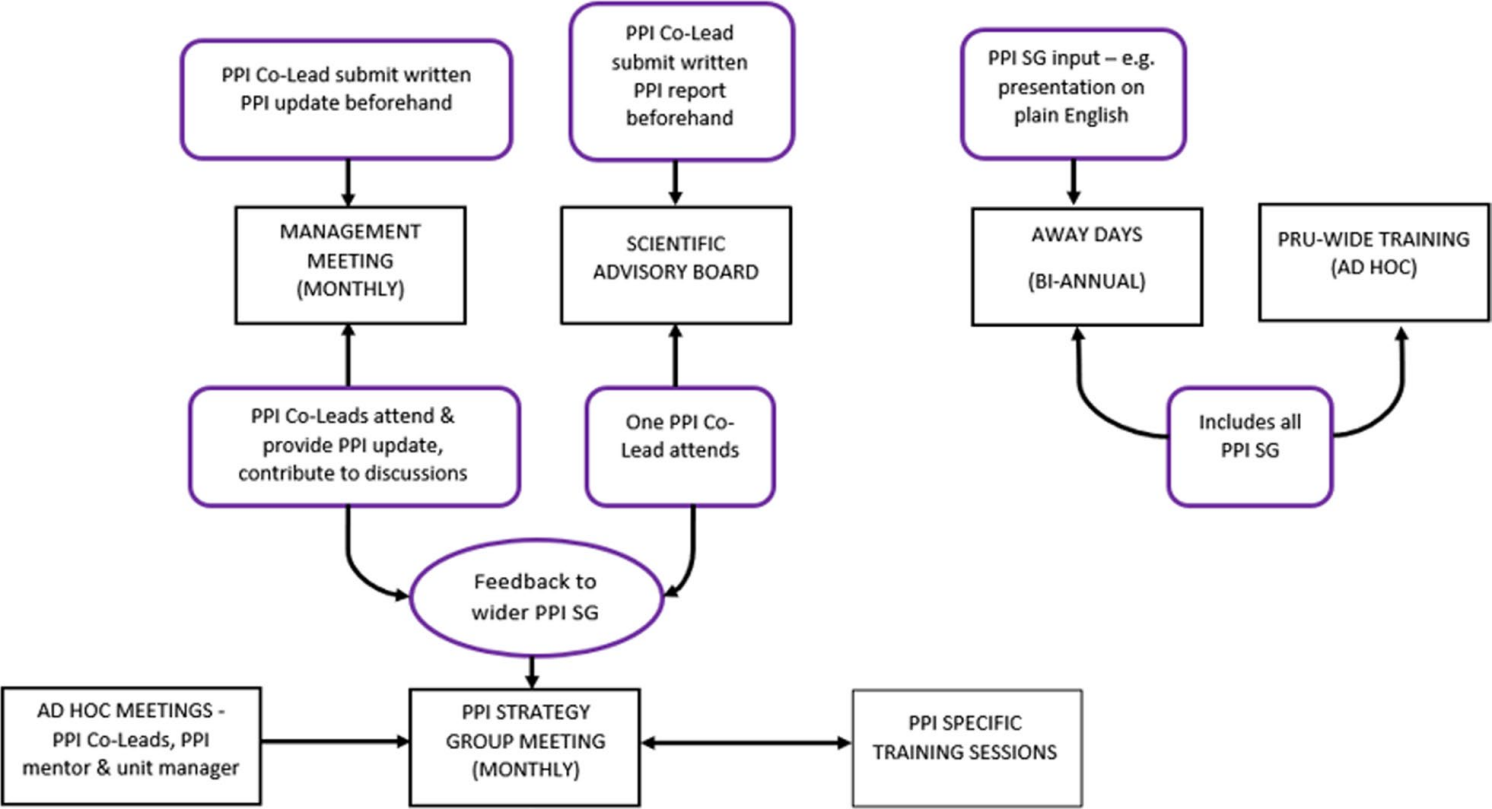
Meeting and training structure and Strategy  Group Involvement

We considered it part of our remit to not only provide training appropriate to the work of the unit but to give members additional skills and knowledge to contribute to the wider PPI. The PPI SG prioritises its own training needs and this is complemented by their inclusion in all PRU-BS training. Training in presentational skills was useful for dissemination of the unit’s work but also gave members confidence and transferable skills. Some examples of the training have included ethics, plain language summaries, presentational skills and video presentations. We are aware that with good funding we are able to pay for training sessions, so where possible we share training sessions with other groups and individuals. The PPI SG run ad hoc PPI training sessions as necessary including presenting on issues of interest to the wider unit. Figure 3 illustrates the PRU-BS meeting and training structures.

## Involvement in PRU-BS projects

**Fig. 4:**
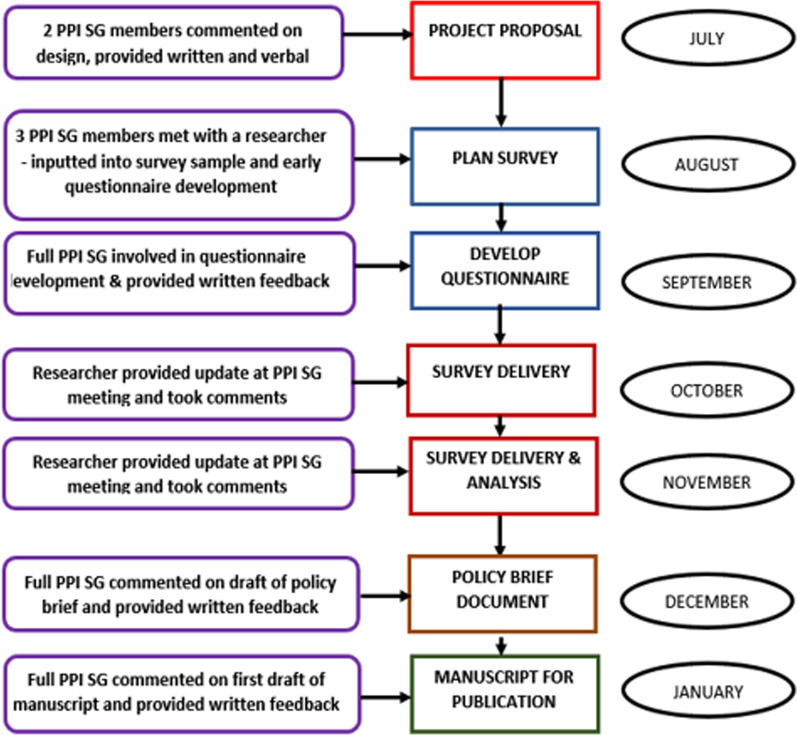
PPI Strategy Group input into flue and Covid-19 vaccine hesitancy

The essential work of the PPI SG is responding to requests for comment and input into projects. Researchers who wish to engage with the group first contact the unit manager and lead and co-lead. They would, if necessary, join the monthly PPI SG meeting to explain their needs and whether a rapid response was required.We demonstrate in Fig. 4 what a typical PPI SG response would look like, in this case a PRU-BS project of influenza and Covid-19 vaccine hesitancy. Here members of the PPI SG were involved in the full project cycle from developing the project proposal to dissemination of the findings.

The researcher on the vaccine project reported that our input had led to key changes at all stages of the research. This reassured the group that we were delivering what was needed and having a positive impact on the work of the unit. As members commented:

"Understanding how people behave and why they do what they do is central to understanding and changing behaviour. This was especially true when we looked at the vaccine project." PPI SG Member

"I liked the challenge of commenting on the questionnaires in the vaccine project. It was rewarding to have positive feedback from the researchers." PPI SG Member

The basic understanding of behavioural science gained from the induction sessions has been reinforced over time through the practical experience of commenting on and discussing projects with researchers. The success of this strategy is apparent in the positive comments by researchers on the work of the Group.

"It was good to get positive feedback and know what we were doing was what was wanted.!" SG member

An ongoing and specific challenge is ensuring meaningful public involvement in project work, when the time to respond to is extremely short. However, the PPI SG have responded well to provide input at short notice.

"I thought that the way we had to respond to requests at short notice would be difficult, but it was easier than I thought, now it is just part of what we do." PPI SG Member

## Where are we now?

The members of the PPI SG are an essential part of the unit and fully involved with every stage of the research cycle – from the inception of the research question through to dissemination—and participation in all other unit activities, such as training and away days. This includes involvement in projects related to Covid-19 [18], shared medical appointments [19], interventions to direct the public to the right health service, and preventing urinary catheter infections. Input from group members into several of these projects has elicited a positive response from researchers which bodes well for the continued integration of the PPI SG with the PRU-BS. The work of the PPI SG is just beginning but the indications are that it is an important part of the unit and enables the PRU-BS to continue to produce cutting edge, world leading research.

Since the advent of Covid-19 all of the group’s work by necessity has been conducted via Zoom, the Voice platform and emails. This does however mean that members from regions outside of the North East who would not always be able to meet face to face could participate more easily.

## Recommendations for creating a PPIE Strategy Group

Research ultimately effects the public in some way. The public, with their unique skills, experiences and insights, gained through their roles as carers, as service users, or in their various occupations need to be involved. The phrase ‘nothing about us without us’ is apt here. Creating a PPI SG for the PRU-BS has been an exciting challenge and there are many lessons learnt. We have drawn nine recommendations based on our experiences.

Point 1: PPI leads need to be involved from the beginning in the formulation of funding applications for units of this kind and given co-applicant status. This provides continuity and commitment from the initial planning to the shaping of ideas and forming a basis for future development.

Point 2: It is important that there is no prioritising of optimism over reality when it comes to PPI. No plan survives intact from its first contact with reality. There is a need to adapt, be flexible and respond to changing circumstances.

Point 3: Establishing an entirely new research unit takes time. Research staff must be recruited and there needs to be a modicum of administrative infrastructure in place before PPI can fully take shape. Establishing a PPI group before there is a clear role for them could result in public members losing interest.

Point 4: It is essential that a PPI lead and co-lead are appointed early to develop a plan of action and consider key documents such as the Terms of Reference and Strategy. On reflection, a great opportunity was missed by not reaching out to other PPI Strategy Groups within the NIHR infrastructure organisations to learn from their experiences. In hindsight this would have had the potential to save time and effort.

Point 5: We adopted a pragmatic approach in drawing up the Terms of Reference and the strategy document and used those already developed by others. However, ideally these needed the input of the wider group. Although there is a chicken and egg situation here, which comes first, the documentation or the appointing of the public group, it is felt that the whole group needs to be involved. This has the advantage that the strategy document can evolve to meet the needs of the PRU-BS and the group, it also gives the group ownership of the document and therefore a greater commitment.

Point 6: While asking individual members of the public with known skills, knowledge and experiences to join a PPI SG is an expedient route, we recognise that advertising more widely for members is a more efficient way of reaching a more diverse group of people. However, a combination of the two has proved effective. In hindsight recruiting the four initial members of the group meant that we could get to know each other and establish a way of working rather than involving seven total strangers. It also meant we had early support and gained experience of managing a group before the number of members increased.

Point 7: In setting up the virtual platform it is important that there is a progressive dialogue with action points which are followed up and regular meetings scheduled to avoid a break in communications and delay. It needs to be stressed that there are many technical issues which are often outside the skill set of the public members, that require the input of professional staff, therefore clarity and good communications are essential. A certain degree of consensus is essential here and an ability to reach a decision, acknowledging that everyone has different skills knowledge and experiences.

Point 8: Smooth effective working is dependent on good relationships between public, managers and researchers, every effort should be made to ensure that this happens. For instance, the inclusion of DG and IS in the management group and the involvement of PPI SG members in the initial projects helped to build and establish relationships with the researchers.

Point 9: Induction of new members into the group is essential to ensure every member has access to the same information regarding the nature and work of the unit. Everyone needs to start from the same level playing field. Meeting face to face with the original four members was a distinct advantage in getting to know each other socially, trying to do so virtually is a challenge although the WhatsApp group set up by one of the PPI SG members acts as a space where we can interact socially.

## Conclusions

In this commentary we have described our experiences of creating a PPI SG and given recommendations we hope will be beneficial to others wishing to do the same. As stated earlier, there is little literature on forming a group outside of the usual PPI for the duration of a single research project. Of the two studies we identified we found distinct differences in selection and training. First, their PPI groups are involved in projects which focus on one issue [13] or condition [14] whereas when recruiting to the PPI SG we had to consider the very diverse nature of the research they would be involved in. Projects could vary from research exploring the uptake of the Covid vaccine to issues related to the implementation of virtual wards. Second, SG members often have to respond rapidly to requests from government. We did not recruit members with experience in a particular condition or topic but needed to select those who met the criteria and demands of the unit. This had implications for training which needed to be wide ranging and comprehensive unlike with PPI on projects that focus on a single theme or condition. In comparison, training for the PPI group on data linkages [13] focused on an explanation of technical terms and information governance. The similarities between creating PPI groups in these different settings and our own was the necessity not only to work closely with researchers and management but also the importance of developing strong relationships. It was apparent in all cases that this did not preclude a professional, indeed robust, relationship. Finally, while the unit’s PPI SG retained many of the same qualities as these other PPI groups, challenges such as ensuring inclusion, being an integral part of the research cycle and working with researchers and others to ensure research is of the very highest standard remained the same. It has been argued that striving to achieve an inclusive group may lead to a mismatch of skills and PPI that is tokenistic [20]. However, in creating our group we recruited members to meet the behavioural science criteria, as described earlier, through a national register of public members. By doing so we attracted a wider pool of applicants and were able to meet our objective of a truly diverse group, with regard to gender, ethnicity and age.

In conclusion, we believe valuable lessons have been learned about the realities and pace of implementing, in a real-world setting, the PPI that is proposed in funding applications. Hopefully our experience will be of value to others embarking on such a venture.

## Data Availability

Not applicable.
